# Effects of rigid and non-rigid image registration on test-retest variability of quantitative [^18^F]FDG PET/CT studies

**DOI:** 10.1186/2191-219X-2-10

**Published:** 2012-03-10

**Authors:** Floris HP van Velden, Paul van Beers, Johan Nuyts, Linda M Velasquez, Wendy Hayes, Adriaan A Lammertsma, Ronald Boellaard, Dirk Loeckx

**Affiliations:** 1 Department of Nuclear Medicine & PET Research, VU University Medical Center, Amsterdam, The Netherlands; 2 Division of Nuclear Medicine and Medical Imaging Center, University Hospital Leuven, Leuven, Belgium; 3 Discovery Medicine and Clinical Pharmacology, Bristol-Myers Squibb Co., Princeton, NJ, USA; 4 Center for Processing Speech and Images, Department of Electrical Engineering, KU Leuven, Leuven, Belgium

**Keywords:** Positron emission tomography (PET), Test-retest variability, Image registration, Non-rigid, Rigid

## Abstract

**Background:**

[^18^F]fluoro-2-deoxy-D-glucose ([^18^F]FDG) positron emission tomography (PET) is a valuable tool for monitoring response to therapy in oncology. In longitudinal studies, however, patients are not scanned in exactly the same position. Rigid and non-rigid image registration can be applied in order to reuse baseline volumes of interest (VOI) on consecutive studies of the same patient. The purpose of this study was to investigate the impact of various image registration strategies on standardized uptake value (SUV) and metabolic volume test-retest variability (TRT).

**Methods:**

Test-retest whole-body [^18^F]FDG PET/CT scans were collected retrospectively for 11 subjects with advanced gastrointestinal malignancies (colorectal carcinoma). Rigid and non-rigid image registration techniques with various degrees of locality were applied to PET, CT, and non-attenuation corrected PET (NAC) data. VOI were drawn independently on both test and retest scans. VOI drawn on test scans were projected onto retest scans and the overlap between projected VOI and manually drawn retest VOI was quantified using the Dice similarity coefficient (DSC). In addition, absolute (unsigned) differences in TRT of SUV_max_, SUV_mean_, metabolic volume and total lesion glycolysis (TLG) were calculated in on one hand the test VOI and on the other hand the retest VOI and projected VOI. Reference values were obtained by delineating VOIs on both scans separately.

**Results:**

Non-rigid PET registration showed the best performance (median DSC: 0.82, other methods: 0.71-0.81). Compared with the reference, none of the registration types showed significant absolute differences in TRT of SUV_max_, SUV_mean _and TLG (p > 0.05). Only for absolute TRT of metabolic volume, significant lower values (p < 0.05) were observed for all registration strategies when compared to delineating VOIs separately, except for non-rigid PET registrations (p = 0.1). Non-rigid PET registration provided good volume TRT (7.7%) that was smaller than the reference (16%).

**Conclusion:**

In particular, non-rigid PET image registration showed good performance similar to delineating VOI on both scans separately, and with smaller TRT in metabolic volume estimates.

## Background

Positron emission tomography (PET) has now been accepted as a valuable tool in oncology, not only for detecting or staging disease and estimating target volumes for radiotherapy purposes, but also for monitoring response to therapy and predicting prognosis [1,2]. To date, [^18^F]fluoro-2-deoxy-D-glucose ([^18^F]FDG) is the most widely used tracer for oncological applications. Especially for monitoring response to therapy, it is likely that quantitative assessment of [^18^F]FDG uptake will become the standard. One practical issue in longitudinal PET/CT studies is that patients are not scanned in exactly the same position. Therefore, baseline volumes of interest (VOI) cannot be reused directly. Reusing baseline VOI are of interest for measuring changes in tracer uptake (response) compared with baseline and for studying changes in uptake heterogeneity [3]. Rigid or non-rigid image registration needs to be applied to enable reuse of baseline VOI on response scans. Rigid image transformation only allows for rotational and translational movements of the entire image, whereas non-rigid image registration allows for any type of local (elastic) deformations. De Moor et al [4] showed that non-rigid image registration of [^18^F]FDG images could be used for easier and faster therapy assessments.

Validating image registration strategies for response assessments poses several problems. Response monitoring requires a scan before therapy (a baseline scan), followed by one or several scans sometime during treatment. Because the interval between these scans can be several weeks, tumours are likely to change in volume and/or tracer uptake due to either treatment or progression of disease. On the other hand, test-retest studies are acquired within a limited time frame, without administration of therapy between scans, so only small differences in metabolic volume and tracer uptake are expected. Image registration strategies that fail to properly register such test and retest scans can be discarded for future use and, therefore, application to test-retest studies should be regarded as a first attempt to validate image registration strategies for oncological response assessment studies.

The purpose of this study was to investigate the impact of various image registration strategies on standardized uptake value (SUV) and metabolic volume test-retest variability (TRT). To this end, test-retest PET/CT data from patients with advanced gastrointestinal malignancies were collected retrospectively in order to evaluate various image registration strategies as candidates for registration of response assessment studies. Previously, effects of intermodality rigid and non-rigid image registration on maximum SUV (SUV_max_) and metabolic tumour volume have been determined for non-small cell lung cancer patients [5]. It was shown that the type of registration had no significant impact on SUV_max_. To the best of our knowledge, this is the first study that reports on the impact of various types of intramodality image registration (with different levels of image cropping and various types of input data) on various quantitative measures derived from the PET scan (and in particular on the repeatability of those measures).

## Methods

### Patient data

Two baseline whole-body [^18^F]FDG PET/CT studies were acquired for 11 subjects (9 male, 2 female; age: 63 ± 11 y (mean ± standard deviation); weight: 90 ± 15 kg) with advanced gastrointestinal malignancies (colorectal carcinoma) at five different sites [6]. Nine patients were scanned on a Biograph PET/CT scanner (CTI/Siemens, Knoxville, TN, USA) and two patients were scanned on a Gemini TF-64 PET/CT scanner (Philips Healthcare, Cleveland, OH, USA). Test and retest studies were performed within 12 days (5.5 ± 3.4 days) of each other. All patients fasted for at least 4 h before scanning and refrained from strenuous activity. Blood glucose levels were obtained for each patient prior to scanning and these levels were within the normal range (5.7 ± 1.4 mmol·l^-1 ^). Patients had received no therapy (chemotherapy, radiotherapy, or surgical treatment) for at least 2 weeks prior to the first baseline [^18^F]FDG PET/CT scan.

A static whole-body emission scan was started 79 ± 20 min after injection of [^18^F]FDG (509 ± 98 MBq). Prior to this emission scan, a low dose CT scan was acquired for attenuation correction purposes (120-130 kVp and 54-133 mAs) during normal breathing. All data were acquired and reconstructed according to local guidelines that were in accordance with recently published guidelines for quantitative [^18^F]FDG PET studies [7,8]. For the Gemini TF-64 PET/CT, PET images were reconstructed onto a 144 × 144 image matrix (voxel size: 4.0 × 4.0 × 4.0 mm) using a row action maximum likelihood algorithm. The corresponding CT images were reconstructed onto a 512 × 512 image matrix with a voxel size of 0.78 × 0.78 × 5.0 mm. For the Biograph PET/CT, PET images were reconstructed onto either 128 × 128 (voxel size: 5.2 × 5.2 × 2.4 mm, n = 2; or 5.3 × 5.3 × 3.4 mm, n = 5) or 168 × 168 (voxel size: 4.1 × 4.1 × 2.0 mm, n = 2) image matrices using an ordered-subsets expectation maximization algorithm. The corresponding CT images were reconstructed onto a 512 × 512 image matrix with a voxel size of 0.98 × 0.98 × 2.4 (n = 2), 0.98 × 0.98 × 2.5 (n = 5) or 0.98 × 0.98 × 4.0 (n = 2) mm. Both attenuation corrected and non-attenuation corrected (NAC) PET images were obtained. After reconstruction, attenuation-corrected PET data were transformed to SUV using:

$$\frac{activity\;\left(kBq/ml\right)}{injected\;dose\;\left(MBq\right)/body\;weight\;\left(kg\right)}$$

All PET and CT data were acquired as part of an ongoing clinical study [6], which was approved by an authorised medical ethical review committee, and informed consent was obtained from each patient prior to inclusion in the study.

### Image registration strategies

Several rigid and non-rigid strategies were attempted, based on various input data:

• PET to PET image registration. This registration type takes functional information into account;

• NAC to NAC image registration, after which the transformation was applied to the PET images. NAC images are not affected by erroneous attenuation correction that might be caused by a possible (small) mismatch between PET and CT;

• CT to CT image registration, after which the transformation was applied to the PET data. This registration takes anatomical information into account. No global mismatches between CT and PET images were observed. The low dose CT scans were downsampled to the PET resolution prior to image registration to increase computational performance and to avoid issues with computer memory;

• CT to CT image registration, after which the transformation was used to initialize PET to PET registration (referred to as CTPET). This registration takes first the anatomical and second the functional information into account. This method was only used for (non-linear) non-rigid transformations, as (linear) rigid CTPET-based image registration would produce identical results to rigid PET-based image registration.

These various types of image registration were applied on:

• Whole-body images as obtained from the PET/CT reconstruction, referred to as 'global';

• Whole-body images as obtained from the PET/CT reconstruction, cropped from the shoulders until just above the bladder, so that they did not include high tracer uptake regions (i.e. the head and bladder). This method is referred to as 'semi-local';

• Whole-body images as obtained from the PET/CT reconstruction, cropped in such a way that they included either only the liver or only the lung region. This method is referred to as 'local'.

In total, 21 different image registration strategies were investigated, summarized in Table 1. In all cases similarity between scans was measured by minimizing summed squared differences (SSD), maximizing normalized cross correlation (NCC) or maximizing mutual information (MI) [9]. For non-rigid PET registration, high uptake regions (regions having a SUV larger than 10, e.g. the bladder or the brain) were limited to a SUV of 10 to enhance performance and to avoid image artefacts. When MI was used, this was applied during (joint) histogram calculations to avoid loss of performance due to the limited number of grey level bins. When NCC and SSD were used, this was applied to images before registration to create temporary images that were used as input for the image registration, thereby increasing proper matching of certain high uptake regions that can vary highly amongst different scans, i.e. the bladder.

**Table 1:** Overview of the registration strategies

	Registration		Input for registration	
**Transformation**	**Program**	**Similarity measure**	**Input data**	**Focus**

Rigid	Elastix	SSD	PET	Global
		NCC		Semi-local
		MI		Local
	RegisRigid	SSD	NAC	Global
		NCC		Semi-local
		MI		Local
Non-rigid	Elastix	SSD	CT	Global
		NCC		Semi-local
		MI		Local
	splineMIRIT	SSD	CTPET^a^	Global
		NCC		Semi-local
		MI		Local

^a^CTPET is used for non-rigid registrations only; *SSD *= summed squared differences; *NCC *= normalized cross correlation; *MI *= mutual information

### Software packages

To confirm independence from implementation, three software packages were used from two different institutes:

• Elastix (UMC Utrecht, The Netherlands) for both rigid and non-rigid image registrations [10];

• splineMIRIT (KU Leuven, Belgium) for non-rigid image registrations [11];

• Regisrigid (KU Leuven, Belgium) for rigid image registrations [4].

In case of Elastix, optimal parameters for the adaptive stochastic gradient descent optimisation method (i.e. step size) were derived for each combination of strategy, similarity measure and type of image registration (rigid or non-rigid) using data from one patient [12]. Four resolution levels were applied, except for rigid PET for which three resolution levels were applied. In the finest resolution level, the control point spacing of the B-spline transformation was set to 16 mm and 32 mm for rigid and non-rigid transformations, respectively. In each resolution level, 32 grey level bins and 2,000 spatial samples were used to compute the mutual information. A maximum of 500 iterations were applied. During registration and optimization the order of B-spline interpolation was set to linear. Prior to any non-rigid registration, a rigid registration was performed. More details on the implementation of Elastix can be found elsewhere [10].

In case of splineMIRIT, stages of resolution and refinements were optimized for each strategy to avoid artefacts and increase performance. In total, 10, 6 or 10 stages of resolutions and refinements were used for non-rigid PET, CT or CTPET registration, respectively. In each resolution level, 32 grey level bins were used to compute the mutual information. No maximum of iterations were applied. Quadratic B-splines have been used for interpolation. No rigid registration was performed prior to non-rigid registration. More details on the implementation of splineMIRIT can be found elsewhere [11].

No optimization or calibration was required for RegisRigid. RegisRigid makes use of a greedy search algorithm and three different resolution levels to obtain translation and rotation parameters [4]. In each resolution level, 20 grey level bins were used to compute the mutual information. No maximum of iterations were applied. During registration and optimization trilinear interpolation was used.

### Data analysis

In total, 24 lesions could be identified that were located in liver (n = 13), lung (n = 10) or bone (n = 1). VOIs were drawn on both test (VOI_test_) and retest (VOI_retest_) scans using a 3 dimensional (semi)-automatic isocontour method at 50% of the maximum pixel value [13]. For each VOI, SUV_max_, mean SUV within a VOI (SUV_mean_), volume of the VOI (metabolic volume) and total lesion glycolysis (TLG, calculated as product of SUV_mean _and metabolic volume) were obtained. For all these parameters, both relative (signed) and absolute (unsigned) TRT values were calculated as the (absolute) difference between the results of the test and retest scans, divided by the mean of these two values. These TRT values were used as a reference.

To validate the image registration techniques, VOI_test _were transformed according to the transformation parameters obtained (resulting in VOI_reg_). Dice similarity coefficients (DSC) were calculated between VOIreg and VOIretest using $DSC\left(X,Y\right)=\frac{2|X\cap Y|}{\left|X|+|Y\right|}$ where |*X*| denotes the volume of VOIreg, |*Y*| the volume of VOIretest, and |*X *∩ *Y*| the overlap between the two volumes. In addition, VOI_reg _were applied to the retest scans to obtain SUV_max_, SUV_mean_, metabolic volume and TLG. To measure the impact on TRT, for each parameter, absolute (unsigned) TRT was calculated as the absolute difference between results of the test scans (using VOI_test_) and those of the retest scans using VOI_reg_, after which these results were compared with the absolute reference TRT data. To assess whether any of the registration strategies showed a systematically different trend compared with the reference, relative (signed) TRT values were also calculated and compared with relative reference TRT values. A two-tailed Wilcoxon signed ranks test was applied to reference TRT values and TRT values obtained after image registration, or between DSC values obtained using Elastix, RegisRigid or splineMIRIT. P-values less than 0.05 were considered significant.

As there is no reference value for DSC, an estimate was made of the largest possible overlap in volumes between both VOI delineated separately on test and retest scans. This number is fictive and, theoretically, can only be achieved when VOI_test _can be folded exactly onto VOI_retest_, retaining its original volume.

## Results

### Computation time

Depending on the registration strategy and level of image cropping, the computation times on a PC with Core 2 Duo 2.53 GHz cpu (Intel, Santa Clara, CA, USA) were around 0.5 to 4 min for Elastix, 1 to 3 min for RegisRigid and 20 to 90 min for splineMIRIT.

### Similarity measures

Table 2 shows median DSC values for various global registration strategies and using several similarity criteria. Overall, NCC provided the highest DSC for PET, NAC and CTPET based image registration strategies and MI for the CT based image registration strategy. In the remainder, only results for these similarity measures (i.e. MI for CT and NCC for other image registration strategies) will be presented.

**Table 2:** Median Dice similarity coefficients (DSC) for various registration strategies and similarity measures

Transformation	Input data	Program	Similarity measure		
			
			SSD	NCC	MI
Rigid	PET	Elastix	0.74	0.71	0.68
		RegisRigid	0.65	0.59	0.54
	NAC	Elastix	0.59	0.59	0.56
		RegisRigid	0.59	0.60	0.60
	CT	Elastix	0.71	0.71	0.72
		RegisRigid	0.72	0.72	0.71

Non-rigid	PET	Elastix	0.81	0.82	0.71
		splineMIRIT	0.77	0.78	0.77
	NAC	Elastix	0.69	0.72	0.21
		splineMIRIT	0.66	0.73	0.74
	CT	Elastix	0.68	0.67	0.68
		splineMIRIT	0.59	0.60	0.68
	CTPET	Elastix	0.82	0.82	0.69
		splineMIRIT	0.77	0.78	-^a^

^a^The program was not able to obtain registrations due to memory issues; *SSD *= summed squared differences; *NCC *= normalized cross correlation; *MI*= mutual information

### Effects of input data on DSC

**Figure 1:**
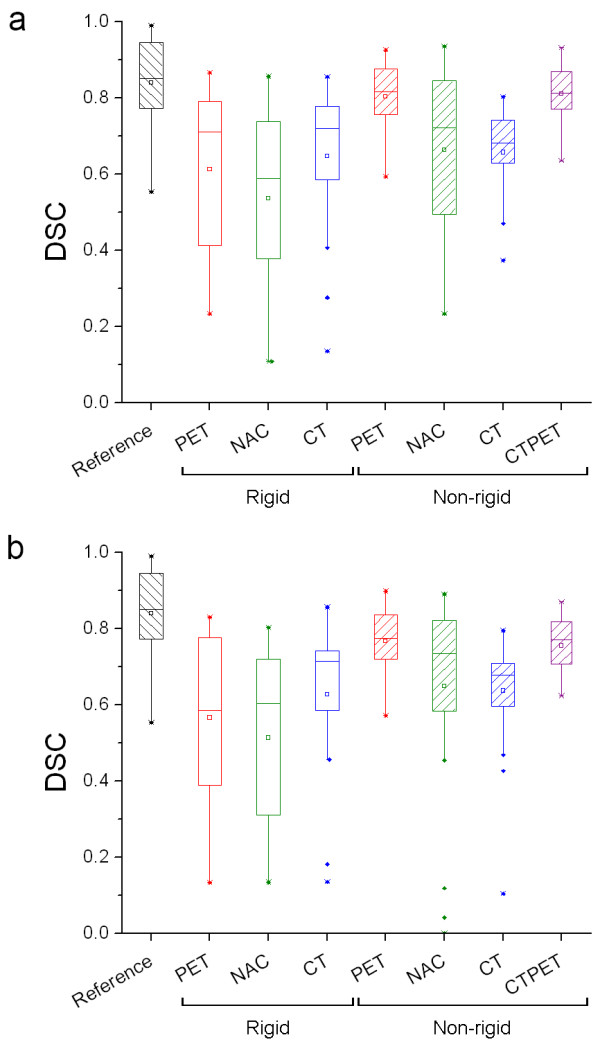
**Effects of input data on DSC**. Box plots of DSC obtained using rigid and non-rigid registration strategies and various input data. DSC values were obtained using (**a**) Elastix or (**b**) RegisRigid and splineMIRIT. Reference shows the largest overlap in volume that could be achieved for VOI delineated on both scans separately. The mean is illustrated by a square, outliers by dots, and minimum and maximum values by crosses.

Figure 1 shows box plots illustrating DSC of global rigid and non-rigid registration strategies using various input data, with corresponding mean, median and range given in Additional file 1: Table S1. Non-rigid image registration outperformed rigid image registration for most input data (23% higher median DSC), except CT (5% lower median DSC). For rigid registration, CT input data provided the highest DSC (median: 0.72 and 0.71 using Elastix and splineMIRIT, respectively), while for non-rigid image registration, both PET and CTPET input data resulted in the highest DSC (median: 0.82 and 0.78 using Elastix and splineMIRIT, respectively). NAC data did not provide an improvement in median DSC for rigid registration strategies and showed more artefacts in the registered images following non-rigid image registration, resulting in an increased number of outliers. Therefore, in the remainder only results using CT, CTPET and PET as input data for registrations will be provided. Both Elastix (Figure 1A) and RegisRigid/splineMIRIT (Figure 1B) provided similar trends and most differences between programs were non-significant (p > 0.12). Elastix, however, showed a small but significant improvement in non-rigid PET and CTPET image registration of 5% in median and 4% in mean DSC values (p < 0.001). Therefore, only data obtained using Elastix will be presented.

### Effects of image cropping on DSC

**Figure 2:**
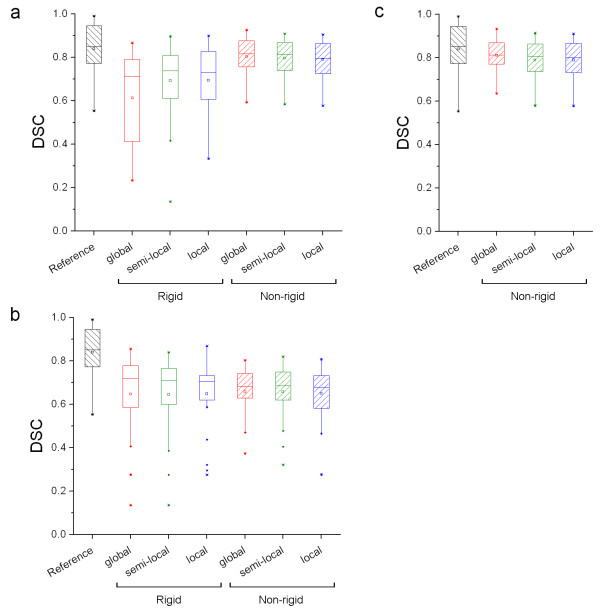
**Effects of image cropping on DSC**. Box plots of DSC obtained using rigid and non-rigid registration strategies and various levels of image cropping. DSC values were obtained using (**a**) PET, (**b**) CT or (**c**) both (CTPET). Reference shows the largest overlap in volume that could be achieved for VOI delineated on both scans separately. The mean is illustrated by a square, outliers by dots, and minimum and maximum values by crosses.

**Figure 3:**
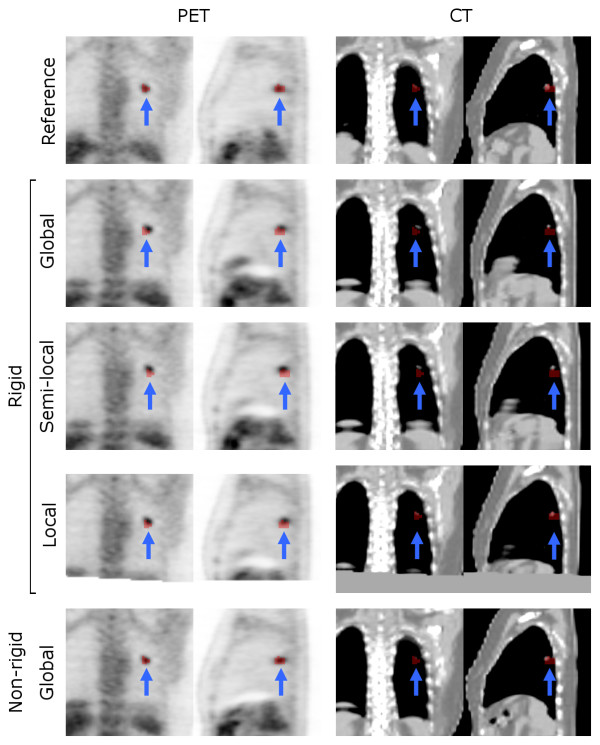
**Example images of one patient with lung lesions**. Example of coronal and sagittal images of one patient with lung lesions, illustrating the effects of various rigid and non-rigid registration strategies using CT or PET as input data. The four lower rows show the test scan registered onto the retest scan (reference, shown on the top row). VOI_retest _is shown in red, indicted by blue arrows. All images are resliced to the same position of VOI_retest _and all images are shown using the same colour scales.

In Figure 2 box plots are shown illustrating the effects of various levels of image cropping on DSC of rigid and non-rigid registration strategies using various input data. Corresponding mean, median and range are given in Additional file 2: Table S2. All changes in performance of semi-local and local compared with global were insignificant (p > 0.05). As various levels of image cropping showed no improvement and an insignificant change in DSC for non-rigid image registration strategies (p > 0.36), cropping in combination with non-rigid image registration will not be considered further. A small, albeit insignificant (p > 0.14), improvement in median DSC values was observed for local rigid image registration compared with global rigid image registration. For global rigid PET registration, 80% of lung lesions showed a DSC of less than 0.50. Using local PET registration, this number decreased to 40%. In case of non-rigid PET registration, however, all lung lesions showed a DSC of more than 0.59. For semi-local rigid PET registration, one subject with a lung lesion showed a decrease in DSC (from 0.38 to 0.14) compared with global and local PET registration (Figure 3). Consequently, only results of local rigid image registration will be reported as an illustration of the effects of cropping on various quantitative measures. Figure 3 also shows a mismatch between CT and PET, causing CT registration to show less overlap between VOI and lesion than PET registration. For rigid CT registration, only 30% of the lung lesions showed a DSC of less than 0.5. Both non-rigid and local CT registration did not change this number.

### Effects of lesion size on DSC

**Figure 4:**
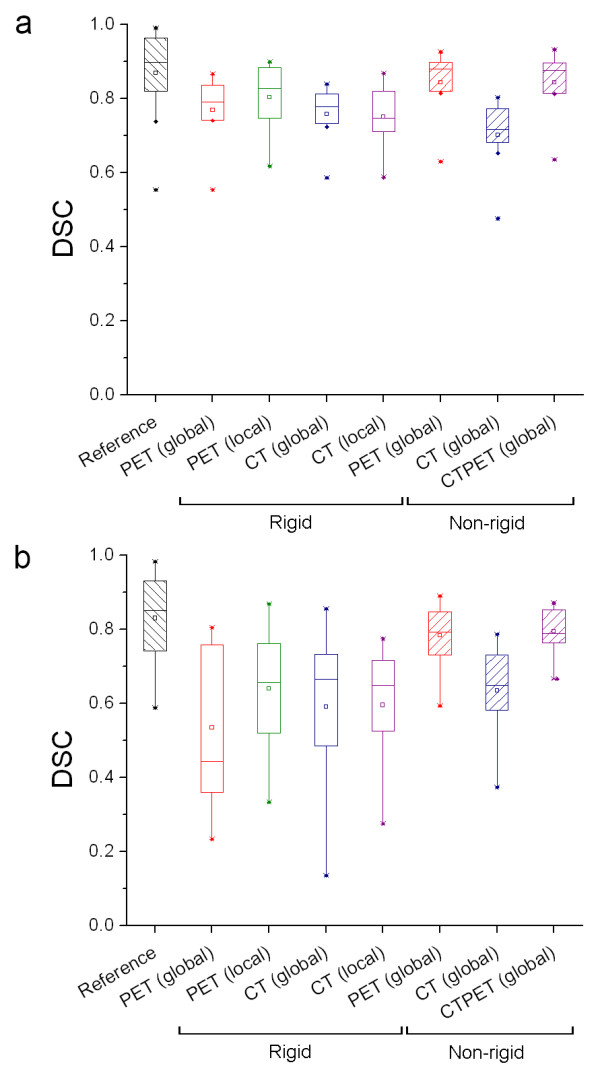
**Effects of lesion size on DSC**. Box plots illustrating the effects of lesion size on DSC obtained using (local) rigid and non-rigid registration strategies. DSC values were obtained from (**a**) large lesions (n = 8; average: 166 ml, range: 48-749 ml) or (**b**) small lesions (n = 16; average: 13 ml, range: 1.1-33 ml). Reference shows the largest overlap in volume that could be achieved for VOI delineated on both scans separately. The mean is illustrated by a square, outliers by dots, and minimum and maximum values by crosses.

Figure 4 shows box plots illustrating the effects of lesion size on DSC of rigid and non-rigid registration strategies using various input data. Corresponding mean, median and range are given in Additional file 3: Table S3. For large lesions (> 35 ml, n = 8, all located in the liver), local rigid PET, non-rigid PET and CTPET registration strategies showed a significantly higher DSC (0.83 to 0.88, respectively) compared to other registration strategies (range: 0.72 to 0.79). For small lesions (n = 16, < 35 ml), however, (local) rigid PET registration and all CT registrations showed a significantly lower DSC (range: 0.44 to 0.65) compared to non-rigid PET and CTPET registration (0.79). In general, ranges of DSC observed for small lesions were larger then those observed for large lesions, except for non-rigid PET and CTPET registrations.

### Effects on TRT of various quantitative PET measures

**Figure 5:**
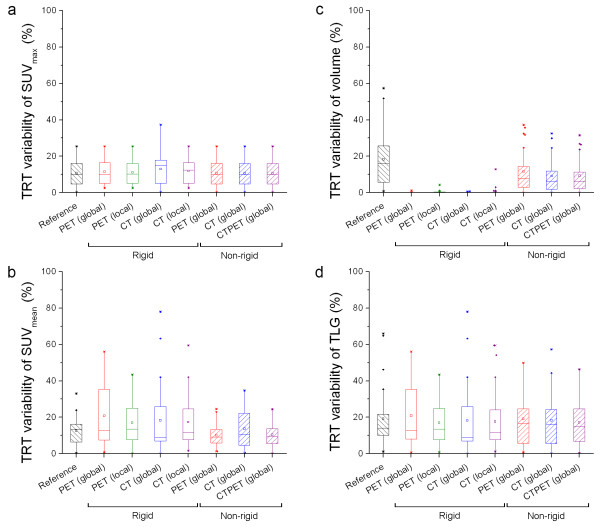
**Effects on absolute TRT of various quantitative PET measures**. Box plots illustrating the effects of (local) rigid and non-rigid registration strategies on the absolute test-retest variability (TRT) of SUV_max _(**a**), SUV_mean _(**b**), metabolic volume (**c**) or total lesion glycolysis (TLG, **d**). These TRT values were obtained using PET, CT or both (CTPET). Reference shows those TRT values that were obtained by delineating VOIs on both scans separately. Lower values are better than higher values. The mean is illustrated by a square, outliers by dots, and minimum and maximum values by crosses.

In Figure 5 box plots are shown illustrating the effects of (local) rigid and non-rigid registration strategies on absolute TRT of various quantitative measures derived from final PET images. TRT of SUV_max _(Figure 5A, Table 3), SUV _mean _(Figure 5B, Table 3) and TLG (Figure 5D, Table 3) showed no significant differences between registration strategies and reference (p > 0.20), except for non-rigid CTPET registration with a significantly lower absolute TRT of SUV_mean _(p < 0.05). Only non-rigid image registration provided an identical TRT of absolute SUV_max _as the reference. Global rigid CT registration showed one subject with a lung lesion that had a higher TRT of absolute SUV_max _(37.3) than other registration strategies (10.3). For all registration strategies, except non-rigid PET registration (p = 0.10), absolute TRT of metabolic volume (Figure 5C, Table 3) were significant lower compared to the reference (p < 0.05). For rigid image registration, some lesions showed small absolute TRT that were larger than zero.

**Table 3:** Absolute values and test-retest variability of various quantitative PET measures obtained with various registration strategies

Measure	Transfor mation	Input data	Focus	Absolute values^a^			Absolute test-retes variability t			Relative test-retest variability		
				
				Mean	Median	Range	Mean	Median	P- value^b^	Mean	Median	P- value^b^
SUV_max_	Reference^c^			17.5	14.5	4.6 - 46.4	10.7	10.0	-	-3.4	-3.5	-
	
	Rigid	PET	Global	17.4	14.5	4.6 - 46.4	11.5	10.0	0.197	-3.4	-3.5	1.000
			Local	17.4	14.5	4.6 - 46.4	11.3	10.2	0.988	-2.8	-3.5	0.855
		CT	Global	17.3	14.5	3.5 - 46.4	13.0	14.8	0.331	-1.0	-3.5	0.900
			Local	17.4	14.5	4.6 - 46.4	11.9	12.5	0.684	-2.2	-3.5	0.768
	
	Non-rigid	PET	Global	17.5	14.5	4.6 - 46.4	10.8	10.0	0.812	-3.4	-3.5	0.136
		CT	Global	17.5	14.5	4.6 - 46.4	10.8	10.0	0.768	-3.4	-3.5	0.410
		CTPET	Global	17.5	14.5	4.6 - 46.4	10.8	10.0	0.812	-3.4	-3.5	0.136

SUVmean	Reference^c^			11.6	9.5	3.1 - 30.5	12.6	13.3	-	-2.7	-5.7	-
	
	Rigid	PET	Global	10.3	8.2	2.2 - 29.5	20.8	12.5	0.229	14.3	9.1	< 0.001^d^
			Local	10.9	8.9	2.2 - 29.5	16.9	13.6	0.264	7.1	2.4	0.037^d^
		CT	Global	10.4	8.2	1.5 - 28.2	18.3	9.0	0.527	12.1	6.3	< 0.001^d^
			Local	10.4	8.3	2.8 - 27.9	17.3	11.8	0.998	11.1	8.7	0.015^d^
	
	Non-rigid	PET	Global	11.1	8.8	3.2 - 29.4	10.2	9.1	0.074	1.2	-0.7	0.001^d^
		CT	Global	10.2	8.3	2.8 - 22.8	13.6	10.5	0.989	8.7	7.0	< 0.001^d^
		CTPET	Global	11.2	9.0	3.2 - 29.4	10.8	10.8	0.049^d^	0.4	-1.4	0.004^d^

Metaboli	Reference^c^			63.9	16.7	1.1 - 749	18.4	16.0	-	0.3	-3.6	-
	
c volume	Rigid	PET	Global	61.6	25.1	1.0 - 711	0.1	0.0	< 0.001^d^	0.0	0.0	0.989
			Local	61.6	25.3	1.0 - 711	0.4	0.0	< 0.001^d^	0.4	0.0	0.900
		CT	Global	61.6	25.3	1.0 - 711	0.1	0.0	< 0.001^d^	-0.1	0.0	0.989
			Local	61.5	25.2	1.0 - 710	0.8	0.0	< 0.001^d^	0.4	0.0	0.900
	
	Non-rigid	PET	Global	66.2	23.2	1.2 - 809	11.7	7.7	0.101	-1.0	-2.4	0.509
		CT	Global	60.1	24.8	1.2 - 686	9.2	6.0	0.034^d^	2.7	1.6	0.584
		CTPET	Global	66.1	23.4	1.0 - 810	11.7	7.2	0.011^d^	2.0	-0.4	1.000

TLG	Reference^c^			713	191	11.1 - 6641	19.2	14.1	-	-2.5	-7.3	-
	
	Rigid	PET	Global	644	168	7.0 - 6051	20.9	12.6	0.812	14.3	9.0	0.007^d^
			Local	662	175	7.0 - 6184	17.1	13.6	0.603	7.3	2.2	0.074
		CT	Global	635	160	4.7 - 5845	18.3	9.1	0.406	12.1	6.2	0.031^d^
			Local	632	162	8.6 - 5834	17.6	11.8	0.049^d^	11.5	8.3	0.015^d^
	
	Non-rigid	PET	Global	709	184	7.1 - 6944	19.1	16.7	0.966	0.1	-0.6	0.967
		CT	Global	607	152	8.7 - 5652	18.4	15.8	0.790	11.3	8.3	0.005^d^
		CTPET	Global	708	181	8.0 - 6957	18.1	14.2	0.768	2.4	0.0	0.345

^a^Units of absolute values, obtained from the retest scan, were g/ml, g/ml, ml and g for SUV_max_, SUV_mean_, metabolic volume and TLG, respectively. ^b^P-values were calculated from data obtained with registration strategy and reference (test-retest variability values that were obtained by delineating VOIs on both scans separately). ^c^Reference illustrates those test-retest variability values that were obtained by delineating VOIs on both scans separately. ^d^Statistically significant difference (P < 0.05); TLG = total lesion glycolysis; VOI = volume of interest

**Figure 6:**
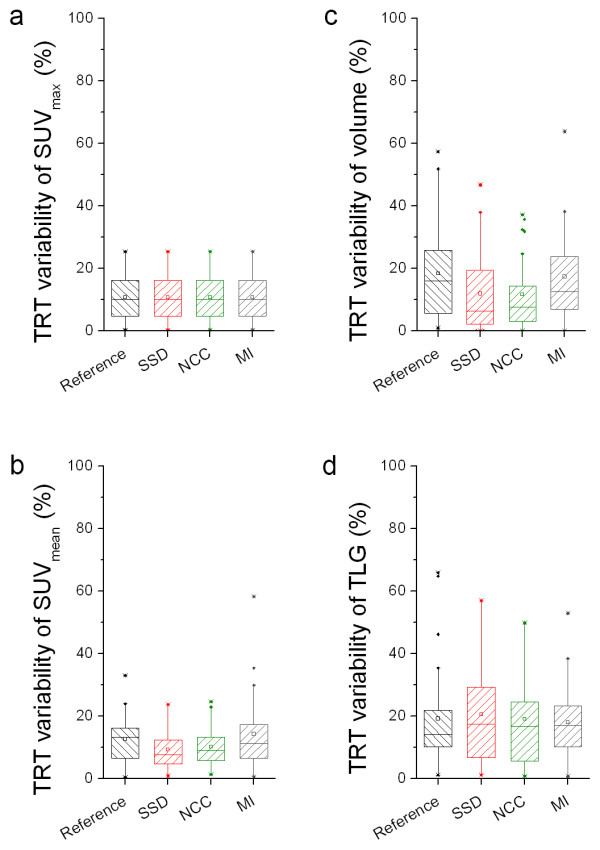
**Effects of similarity measures on absolute TRT of various quantitative measures**. Box plots illustrating the effects of similarity measures derived using non-rigid PET registration on the absolute test-retest variability (TRT) of SUV_max _(**a**), SUV_mean _(**b**), metabolic volume (**c**) or total lesion glycolysis (TLG, **d**). Reference shows those TRT values that were obtained by delineating VOIs on both scans separately. Lower values are better than higher values. The mean is illustrated by a square, outliers by dots, and minimum and maximum values by crosses.

Figure 6 shows effects of similarity measures on absolute TRT of various quantitative measures derived for non-rigid PET registration. Both SSD and NCC showed lower TRT of absolute volume and SUV_mean _than MI. These performance differences corresponded with the results for similarity presented in Table 2.

**Figure 7:**
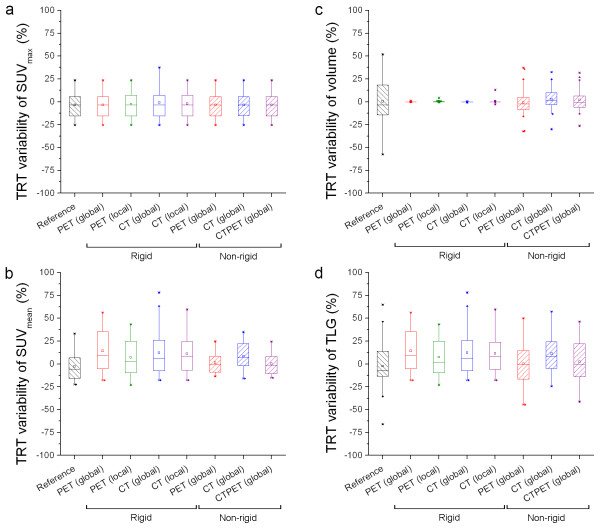
**Effects on relative TRT of various quantitative PET measures**. Box plots illustrating the effects of (local) rigid and non-rigid registration strategies on the relative test-retest variability (TRT) of SUV_max _(**a**), SUV_mean _(**b**), metabolic volume (**c**) or total lesion glycolysis (TLG, **d**). These TRT values were obtained using PET, CT or both (CTPET). Reference shows those TRT values that were obtained by delineating VOIs on both scans separately. Values close to zero are better than higher values. The mean is illustrated by a square, outliers by dots, and minimum and maximum values by crosses.

Figure 7 shows box plots illustrating the effects of (local) rigid and non-rigid registration strategies on relative TRT of various quantitative PET measures. For relative TRT of SUV_mean _(Figure 7B, Table 3), all registrations showed a significant higher median value compared to the reference (p < 0.05). Only non-rigid CTPET and PET registrations showed a negative median value (-1.4 and -0.7%, respectively) in line with the reference (-5.8%). Other registration strategies showed a small positive relative median TRT (range: 2.4-9.1%). This was also reflected in relative TRT of TLG (Figure 7D, Table 3), where CTPET and PET registrations were the only registration strategies that showed no significant differences compared to the reference (p > 0.35). For relative TRT of volume (Figure 7C, Table 3) and SUV_max _(Figure 7A, Table 3) similar trends were observed as for absolute TRT, except that for all registration strategies the differences in metabolic volume were insignificant compared to the reference (p > 0.51).

## Discussion

This study investigates the impact of various image registration strategies on test-retest variability of SUV and metabolic volume derived from repeat [^18^F]FDG PET scans. The main purpose of the study was to identify image registration strategies that can serve as candidates for registration of repeat [^18^F]FDG PET scans in order to monitor response to treatment. To the best of our knowledge, this is the first study that reports on the impact of various types of image registration (with different levels of image cropping and various types of input data) on quantitative measures derived from [^18^F]FDG PET scans. Note that this study did not focus on the accuracy of the entire registered images, but on the accuracy of the registered baseline VOIs only. The idea is that not the images but the VOIs will be transformed, such that tracer uptake quantification will always be performed on the original (i.e. non-transformed) images. In this setting, accurate and precise VOI transformation are most important. However, all registered images were checked visually for image artefacts that might have resulted from (non-rigid) image registration. Occasionally, during optimization and calibration of the registration strategies slightly higher DSCs then those reported in this paper were observed for some patients when other registration parameters were used. However, the use of these parameters was considered not feasible for reuse of baseline VOIs due to (severe) image artefacts that were observed in the registered images. Only those parameters were used that showed a high DSC but did not show image artefacts. Despite that the parameters have been chosen carefully, recalibration of the registration strategies might be required for other purposes (other type of studies or tracers) and final registration results should always be supervised (i.e. visually checked). Two independent software packages were used to investigate the various registration strategies. Results were very similar (Figure 1), although the small improvement in DSC values obtained with Elastix was significant.

Consistent with a previous study [5] showing that the type of intermodality image registration had no significant impact on SUV_max_, the present study showed that the type of intramodality registration had no significant impact on absolute TRT of SUV_max _compared with delineating VOIs separately on both scans. The same was true for SUV_mean _(except for CTPET registration) and TLG. Only for absolute TRT of metabolic volume, significant lower values were observed for all registration strategies when compared to delineating VOIs separately, except for non-rigid PET and CTPET registrations. Rigid image registration does not allow any changes in volume, so in theory this should always be zero. Some lesions, however, showed small non-zero TRT values due to small sampling errors or VOIs that were moved partly outside the image borders after registration.

For relative TRT values, similar trends were observed. However, the type of intramodality registration had no significant impact on relative TRT of metabolic volume. In addition, for relative TRT of SUV_mean _and TLG, significantly higher values were found for all registration strategies, except for relative TRT of TLG obtained using local rigid PET, and non-rigid PET and CTPET.

For most lesions, CT registration provided accurate results. As illustrated in Figure 3, however, some small lung lesions showed small misalignments between PET and CT that could have been caused by respiratory motion. This resulted in a poorer performance of CT registration for these lesions. In these cases, performance of CT image registration could probably be improved by using respiratory gating [14] or intermodality image registration to correct for small residual misalignments between CT and PET [15,16]. In general, performance of CT registration might be improved by using the original CT images that were not downsampled to the PET resolution. Van Herk et al [17] showed that reducing pixel resolution has little effect on performance of rigid CT registration and can be used to speed up the algorithm without loss of accuracy. However, this has yet to be shown for non-rigid CT registrations. NAC image registration was investigated, as (attenuation corrected) PET images could potentially contain errors due to faulty attenuation correction resulting from respiratory motion or from small mismatches between CT and PET. This type of registration, however, provided poorer results than the other registration strategies and, therefore, NAC image registration cannot be recommended.

Despite a small misalignment of a few small lung lesions and the relatively poorer DSC performance compared with PET image registration, CT image registration might still be a good candidate for certain response monitoring studies. Disagreements between CT or PET and clinical response have been observed [18]. When it is of interest to reuse the baseline VOI without changes in volume or shape, i.e. to study changes of [^18^F]FDG uptake within the anatomical volume [3], then CT image registration could be of interest, because (local) rigid CT image registration showed good similarity (median DSC: 0.72) with no change in absolute volume TRT.

Using various levels of image cropping did not show an effect on non-rigid image registration. Roels et al [19] suggested using more local non-rigid image registration to minimize effects of high uptake regions such as the bladder in patients with rectal cancer. In the present study, however, effects of these regions were already minimized by setting the maximum allowed SUV to 10. This could explain why local non-rigid image registrations did not show an improvement in performance.

Rigid PET image registration showed poor results for most lung lesions. In contrast, non-rigid PET and CTPET registrations showed good performance for all types of lesions, and they provided high similarity and similar trends as delineating VOI separately. In addition, CTPET provided significantly lower absolute TRT of volume and SUV_mean_. Nevertheless, as CTPET requires two non-rigid registrations (first CT, followed by PET), non-rigid PET image registration is preferred. For both non-rigid PET and CTPET registrations, DSC was somewhat lower for small lesions (0.79) compared to that for large lesions (0.88). Partial volume effects may be responsible for this. Differences in quantitative measures obtained using non-rigid PET image registration were not significantly different from those obtained by drawing VOI independently on both scans (except for relative TRT in SUV_mean_). This may suggest that there is no additional benefit in using non-rigid image registration. Use of non-rigid image registration, however, will make data analysis easier and faster, because manual search for lesions in the retest scan can be avoided [4]. In addition, drawing VOI on both scans independently is not perfect either, because it still shows an absolute volume TRT of 14%. Although more sophisticated tumour delineation methods may lower this value [13], a significantly smaller absolute volume TRT was observed for non-rigid PET image registration (7.7%). Therefore, in agreement with previous findings [4], non-rigid image registration may be a good alternative for obtaining accurate VOI in response monitoring studies. These results are also consistent with a previous study [16] showing that non-rigid intermodality image registration is a significant improvement over rigid registration for fusion between [^18^F]FDG PET and CT. The present study should primarily be seen as a first attempt to exclude those registration strategies that perform poorly in cases without change in metabolic volume or tracer uptake. However, because tumours are likely to change in metabolic volume and/or tracer uptake during treatment, the remaining registration strategies need to be validated in clinical response monitoring studies.

## Conclusion

Most registration types showed no significant differences in absolute test-retest variability of SUV_max_, SUV_mean _and TLG compared with delineating VOI separately on both scans. In particular, non-rigid PET image registration showed good performance similar to delineating VOI on both scans separately, and with smaller absolute TRT for volume estimates.

## Competing interests

The department of Nuclear Medicine & PET Research of the VU University Medical Center was financially sponsored by Bristol-Myers Squibb Co. for participating in the ongoing clinical study and for providing consultancy. Dirk Loeckx was a postdoctoral fellow of the Research Foundation - Flanders (FWO) until April 2011. He is now co-founder and director of icoMetrix (Leuven, Belgium).

## Authors' contributions

FHPvV performed data analysis and data interpretation, implemented some of the tools to perform image registrations, contributed in the study design and is the main author of the manuscript. PvB performed data analysis and data interpretation. JN implemented RegisRigid, contributed to the intellectual content (supervision) and critically reviewed manuscript. LMV provided PET image data and reviewed the manuscript. WH provided/collected PET image data and reviewed the manuscript. AAL critically reviewed the manuscript and approved the final content of the manuscript. RB performed a study design, supervised the project and reviewed and approved the final content of the manuscript. DL implemented splineMIRIT, contributed to the intellectual content (supervision) and critically reviewed manuscript. All authors reviewed the collected data and interpretation, provided feedback for further research during the study and approved the final submitted version of this manuscript.

## Supplementary Material

Additional file 1 **Table S1**. Mean, median and range Dice similarity coefficients (DSCs) for various registration strategies and programs. Click here for file

Additional file 2 **Table S2**. Mean, median and range for Dice similarity coefficients (DSCs) obtained with various registration strategies. Click here for file

Additional file 3 **Table S3**. Mean, median and range of Dice similarity coefficients (DSCs) obtained with various registration strategies for small (average: 13 ml, range: 1.1-33 ml) and large (average: 166 ml, range: 48-749 ml) lesions. Click here for file
