# Supplementary material for: Effects of rigid and non-rigid image registration on test-retest variability of quantitative [18F]FDG PET/CT studies
Source: EJNMMI Res. 2012 Mar 10;2:10. doi: 10.1186/2191-219X-2-10 (PMC3349514; doi:10.1186/2191-219X-2-10)
Supplement: Additional file 1 — Table S1. Mean, median and range Dice similarity coefficients (DSCs) for various registration strategies and programs. [file 2191-219X-2-10-S1.PDF]

Additional Table 1: Mean, median and range Dice similarity coefficients (DSCs) for various registration strategies and programs.

Transformation	Input data	Program	Mean	Median	Range	P-value[a]
Reference			0.84	0.85	0.55 – 0.99	-
Rigid	PET	Elastix	0.61	0.71	0.23 – 0.87	0.121
		RegisRigid	0.57	0.59	0.13 – 0.83	
	NAC	Elastix	0.54	0.59	0.11 – 0.86	0.241
		RegisRigid	0.51	0.60	0.13 – 0.80	
	CT	Elastix	0.65	0.72	0.14 – 0.86	0.128
		RegisRigid	0.63	0.71	0.14 – 0.86	
Non-rigid	PET	Elastix	0.80	0.82	0.59 – 0.93	<0.001[b]
		splineMIRIT	0.77	0.78	0.57 – 0.90	
	NAC	Elastix	0.66	0.72	0.23 – 0.94	0.584
		splineMIRIT	0.65	0.74	0.00 – 0.89	
	CT	Elastix	0.66	0.68	0.37 – 0.80	0.491
		splineMIRIT	0.64	0.68	0.10 – 0.79	
	CTPET	Elastix	0.80	0.82	0.55 – 0.93	<0.001[b]
		splineMIRIT	0.77	0.78	0.62 – 0.87	

[a]P-values were calculated from data obtained with Elastix and RegisRigid or splineMIRIT
[b]Statistically significant difference ($P < 0.05$)
