# Supplementary material for: Effects of rigid and non-rigid image registration on test-retest variability of quantitative [18F]FDG PET/CT studies
Source: EJNMMI Res. 2012 Mar 10;2:10. doi: 10.1186/2191-219X-2-10 (PMC3349514; doi:10.1186/2191-219X-2-10)
Supplement: Additional file 2 — Table S2. Mean, median and range for Dice similarity coefficients (DSCs) obtained with various registration strategies. [file 2191-219X-2-10-S2.PDF]

Additional Table 2: Mean, median and range for Dice similarity coefficients (DSCs) obtained with various registration strategies.

Transformation	Input data	Focus	Mean	Median	Range
Reference			0.84	0.85	0.55 – 0.99
Rigid	PET	Global	0.61	0.71	0.23 – 0.87
		Semi-local	0.69	0.74	0.14 – 0.90
		Local	0.69	0.73	0.33 – 0.90
	CT	Global	0.65	0.72	0.14 – 0.86
		Semi-local	0.65	0.71	0.14 – 0.84
		Local	0.65	0.71	0.28 – 0.87
Non-rigid	PET	Global	0.80	0.82	0.59 – 0.93
		Semi-local	0.80	0.81	0.58 – 0.91
		Local	0.79	0.79	0.58 – 0.90
	CT	Global	0.66	0.68	0.37 – 0.80
		Semi-local	0.66	0.69	0.32 – 0.82
		Local	0.65	0.68	0.28 – 0.81
	CTPET	Global	0.80	0.82	0.55 – 0.93
		Semi-local	0.79	0.80	0.58 – 0.91
		Local	0.79	0.81	0.57 – 0.90
