# Supplementary material for: Effects of rigid and non-rigid image registration on test-retest variability of quantitative [18F]FDG PET/CT studies
Source: EJNMMI Res. 2012 Mar 10;2:10. doi: 10.1186/2191-219X-2-10 (PMC3349514; doi:10.1186/2191-219X-2-10)
Supplement: Additional file 3 — Table S3. Mean, median and range of Dice similarity coefficients (DSCs) obtained with various registration strategies for small (average: 13 ml, range: 1.1-33 ml) and large (average: 166 ml, range: 48-749 ml) lesions. [file 2191-219X-2-10-S3.PDF]

Additional Table 3: Mean, median and range of Dice similarity coefficients (DSCs) obtained with various registration strategies for small (average: 13 ml, range: 1.1 – 33 ml) and large (average: 166 ml, range: 48 – 749 ml) lesions.

Transformation	Input data	Focus	Small lesions (n=16)			Large lesions (n=8)		
			Mean	Median	Range	Mean	Median	Range
Reference			0.83	0.85	0.89 – 0.98	0.86	0.89	0.55 – 0.99
Rigid	PET	Global	0.53	0.44	0.23 – 0.81	0.77	0.79	0.55 – 0.87
		Local	0.64	0.66	0.33 – 0.87	0.80	0.83	0.62 – 0.90
	CT	Global	0.59	0.67	0.13 – 0.86	0.76	0.78	0.59 – 0.84
		Local	0.60	0.65	0.28 – 0.77	0.75	0.74	0.59 – 0.87
Non-rigid	PET	Global	0.78	0.79	0.59 – 0.89	0.84	0.88	0.63 – 0.93
	CT	Global	0.64	0.65	0.37 – 0.79	0.70	0.72	0.48 – 0.80
	CTPET	Global	0.79	0.79	0.67 – 0.87	0.84	0.88	0.64 – 0.93
